# Careful Interpretation of Positron Emission Tomography Findings in Gastric Schwannomas: A Case Report

**DOI:** 10.7759/cureus.86827

**Published:** 2025-06-26

**Authors:** Hironori Tanaka, Shoji Oura, Hitomi Matsuki, Yurie Kitano, Naoki Kataoka

**Affiliations:** 1 Department of Gastroenterology, Kobe Tokushukai Hospital, Kobe, JPN; 2 Department of Surgery, Kishiwada Tokushukai Hospital, Kishiwada, JPN

**Keywords:** antoni a, gastric schwannoma, high suv max value, infection, pet

## Abstract

Positron emission tomography (PET) is a valuable tool for determining whether tumors are benign or malignant; however, it may occasionally lead to overdiagnosis or underdiagnosis. A 39-year-old woman was incidentally detected with her gastric mass and regional lymph node swelling on computed tomography (CT). Gastro-endoscopy showed a submucosal tumor protruding into the gastric lumen with two overlying ulcers. PET showed a maximal standard uptake (SUVmax) value of 11 in the submucosal gastric tumor but no uptake in the enlarged regional lymph nodes. Despite the absence of malignant cells in the biopsy specimen, the large tumor size, protruding growth pattern into the gastric lumen, avid radiotracer uptake, regional lymph node swelling, and the patient’s young age led us to treat the gastric submucosal tumor not with tumorectomy, but with distal gastrectomy followed by D1 lymphadenectomy to avoid undertreatment. Postoperative pathological study showed that the gastric tumor was an Antoni A-type schwannoma, had intra-tumoral infection, and had no malignant findings in the dissected regional lymph nodes. Physicians should note that gastric schwannoma can have a high SUVmax value and may be overevaluated as a possible malignancy.

## Introduction

Gastric submucosal tumors, such as gastrointestinal stromal tumors (GISTs) [1] and malignant lymphomas [2], are generally covered with normal gastric mucosa and rarely develop any symptoms until they grow large enough to block the passage of food through the stomach or to oppress or invade surrounding organs. Unlike GISTs, benign gastrointestinal peripheral nerve sheath tumors, i.e., schwannomas, are very rare and also do not develop any symptoms in their early phases [3]. In addition, malignant peripheral nerve sheath tumors [4] are extremely rarely observed in the stomach, are often seen in young patients, and exclusively show dismal clinical outcomes.

Physicians can easily approach the stomach using endoscopes and pathologically evaluate not only gastric cancers but also submucosal tumors. Non-epithelial tumors, however, often have heterogeneity similar to epithelial tumors. General surgeons, therefore, sometimes overtreat tumors to avoid undertreatment even when they show benign findings on preoperative pathological examinations. In addition, gastric submucosal tumors rarely develop infections, but when present, these can complicate their diagnosis and treatment.

We herein report a gastric schwannoma overtreated with distal gastrectomy and regional node dissection due to overevaluation of image findings.

## Case presentation

**Figure 1:**
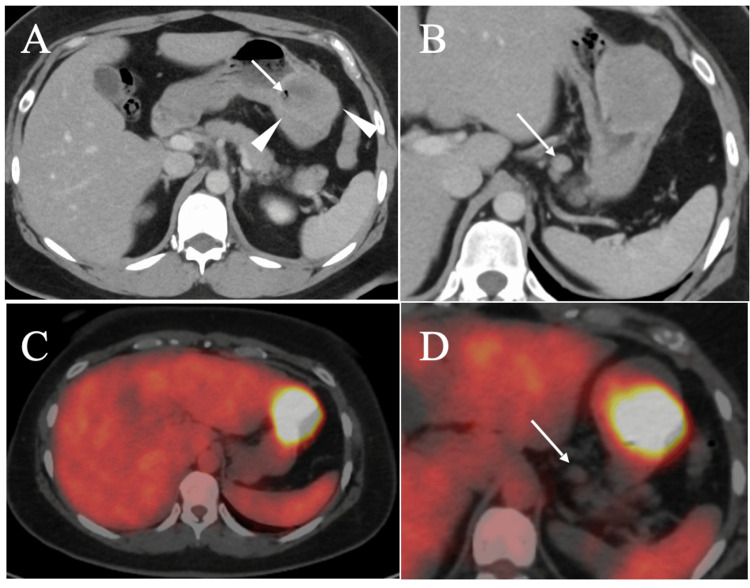
Computed tomography (CT) and positron emission tomography/CT (PET/CT) findings. (A) CT showed a gastric mass with biphasic patterns (arrowheads). Small air (arrow) was observed in the gastric mass. (B) A slightly swollen lymph node was observed around the stomach (arrow). (C) PET/CT showed an avid radio tracer uptake in the gastric tumor. (D) PET/CT showed no radio tracer uptake in the regional node (arrow).

A 39-year-old woman was referred to our hospital for the evaluation and treatment of hypertension. Computed tomography (CT) performed to investigate the cause of hypertension incidentally revealed a heterogeneous 5 cm gastric mass on the anterior wall of the stomach body (Figure 1A), along with regional lymph node swelling (Figure 1B).

**Figure 2:**
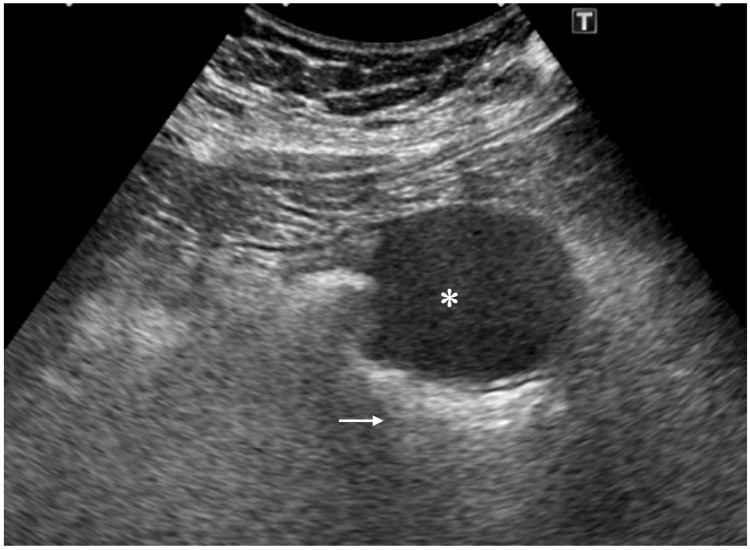
Ultrasound findings showing a mass (asterisk) with clear borders, homogenous and very low internal echoes, and enhanced posterior echoes (arrow).

Ultrasound showed a well-demarcated mass with very low internal echoes and enhanced posterior echoes (Figure 2).

**Figure 3:**
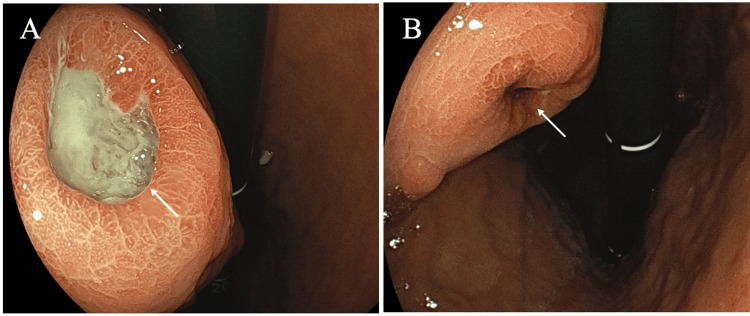
Endoscopic findings. Two ulcers were observed in the gastric mucosa just above the submucosal tumor. The anal side ulcer (A, arrow) was larger than the oral side one (B, arrow) and was filled with mucus-like liquid.

Gastro-endoscopy showed a presumed submucosal tumor protruding into the gastric lumen with both a negative cushion sign and two ulcers just above the tumor (Figure 3).

**Figure 4:**
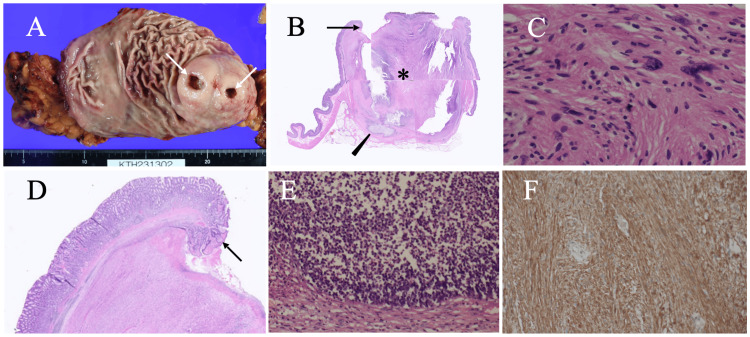
Pathological findings. (A) Two ulcers (arrows) were observed on the tumor. (B) Low-magnification view showed no central necrosis (asterisk), no cystic parts, and a presumed abscess (arrowhead) just beneath the anal-side ulcer. (C) High-magnification view showed spindle cells growing in a palisading fashion. (D) High-magnification view showed normal gastric mucosa (arrow) at the ulcer edges. (E) High-magnification view of the abscess part (B, arrowhead) showed numerous neutrophils. (F) Immunostaining showed that the spindle cells were diffusely positive for S100.

The anal side ulcer was larger than the oral side one and was filled with mucus-like liquid. Positron emission tomography (PET) showed a maximum standard uptake value (SUVmax) of 11 in the gastric submucosal lesion (Figure 1C) but no uptake in the regional swollen lymph nodes, such as para-gastric nodes (Figure 1D). Blood test showed no remarkable abnormalities, including various tumor markers such as carcinoembryonic antigen (CEA), carbohydrate antigen 19-9 (CA19-9), soluble interleukin-2 receptor (s-IL2R), and CA125. An endoscopic biopsy was performed on the tumor through the oral-side ulcer, and pathological examination revealed spindle cells growing in a complex pattern. Immunostaining, however, showed that the tumor was positive for S100 and negative for CD117, αSMA, and desmin [3,5,6], leading to the diagnosis of gastric schwannoma. Although no obvious malignant cells were found in the biopsy specimen, the large tumor size, protruding growth into the gastric lumen, avid radiotracer uptake, regional lymph node swelling, and the patient’s young age led us to treat the gastric tumor not with simple tumorectomy, but with distal gastrectomy and limited regional lymph node dissection (D1 lymphadenectomy), followed by Roux-en-Y reconstruction. Postoperative pathological study showed that the tumor, 60 mm in size, had two ulcers (Figure 4A) just above the anterior edges of the polygonal submucosal tumor (Figure 4B), compact and elongated spindle cells with nuclear palisading (i.e., Antoni A areas; Figure 4C), normal gastric mucosa at the ulcer edges (Figure 4D), an abscess at the bottom of the anal-side ulcer (Figure 4E), diffuse positivity for S100 (Figure 4F), and a Ki-67 labeling index of 8%. 

The patient was discharged on the 10th day after operation without any complications, has been well for 20 months, and is scheduled for annual endoscopic follow-up.

## Discussion

Laparoscopic surgery is a feasible therapeutic option and needs only simple techniques for gastric schwannoma removal, particularly when the tumors are not too large, exhibit an exophytic growth pattern, and are located on the anterior wall of the stomach body [7]. In this case, the large schwannoma protruded into the gastric lumen and required much more complicated surgical procedures than those for exophytic small gastric schwannomas. We, therefore, operated on the patient using similar surgical techniques to those for gastric cancer despite the lack of pathological malignant findings in the endoscopic biopsy specimen.

The high SUVmax value of 11 on PET was another factor for us to select the partial gastrectomy and limited regional node dissection. It is well known that the SUVmax value is useful for judging whether the target lesion either to be benign or malignant. High SUVmax values ​​between the upper cutoff limit and 10 are often observed even in benign tumors or inflammation. Conversely, very few benign tumors and inflammation show SUVmax values higher than 10. Wang et al., however, reported that gastrointestinal schwannomas showed higher SUVmax values than those in other site schwannomas [8]. Antoni A schwannomas, i.e., cellular schwannomas, in particular, can exhibit higher SUVmax values than Antoni B schwannomas [9]. We cannot exclude the possibility that intratumoral infection may have further increased the maximum SUV value. This speculation easily proves to be wrong by the fact that the SUVmax values ​​of the swollen regional lymph nodes, presumably caused by infection, were within normal ranges.

Submucosal tumors are extremely rarely complicated by infection. Schwannomas, however, are more likely to have cystic components [10] than other submucosal tumors such as GISTs and malignant lymphomas. We, therefore, cannot exclude the possible infection of the cystic part of the schwannoma through the overlying ulcer. No cystic components, however, were found pathologically in this case. It, therefore, remains uncertain why intratumoral infection occurred in this case.

Two ulcers were seen on the gastric mucosa at the anterior edges of the polygonal tumor. If central necrosis had led to the ulcer formation, it would have generated only one ulcer rather than two. Furthermore, if the ulcer had been formed due to tumor cell infiltration, the ulcer edge would have been bordered by tumor cells. Ulcer margins, however, were bordered by normal gastric mucosa. It, therefore, is reasonable to judge that the two ulcers did not develop as a result of tumor growth, but rather occurred mechanically by the food passage over the submucosal polygonal tumor.

Non-epithelial malignant tumors, i.e., sarcomas, including malignant nerve sheath tumors, very rarely develop lymph node metastasis, but they do not completely lack lymph node metastasis. Because intra-tumoral infection was first confirmed after the pathological examination of the resected specimen, it was difficult for us to preoperatively rule out that the lymph node enlargement was caused not by the possible malignant tumor but by the intratumoral infection. Therefore, we should have performed a frozen section of the enlarged lymph nodes and proceeded with invasive surgery only if malignant findings were confirmed, as was done in this case.

The Ki-67 labeling index in this case was 8%, slightly higher than that of GISTs [11]. However, regardless of the sites of origin, schwannomas are benign disorders and hardly develop local and distant recurrence when completely resected. We, therefore, plan to continue performing annual endoscopic follow-ups both for local recurrence checks and medical checkups.

## Conclusions

Gastric schwannomas, although benign, can show high SUVmax values and can cause enlargement of regional lymph nodes if the tumors get infected through the overlying ulcer. Therefore, general surgeons should avoid overtreatment of gastric schwannomas based only on high SUVmax values.
